# Perineuronal Nets in the Prefrontal Cortex of a Schizophrenia Mouse Model: Assessment of Neuroanatomical, Electrophysiological, and Behavioral Contributions

**DOI:** 10.3390/ijms222011140

**Published:** 2021-10-15

**Authors:** Razia Sultana, Charles Brady Brooks, Amita Shrestha, Olalekan Michael Ogundele, Charles Chulsoo Lee

**Affiliations:** Department of Comparative Biomedical Sciences, LSU School of Veterinary Medicine, Baton Rouge, LA 70803, USA; cbrooks1@vetmed.lsu.edu (C.B.B.); shrestha1@lsu.edu (A.S.); ogundele@lsu.edu (O.M.O.)

**Keywords:** schizophrenia, DISC1 mutation, perineuronal nets, chondroitinase digestion, behavioral deficits

## Abstract

Schizophrenia is a neurodevelopmental disorder whose etiopathogenesis includes changes in cellular as well as extracellular structures. Perineuronal nets (PNNs) associated with parvalbumin-positive interneurons (PVs) in the prefrontal cortex (PFC) are dysregulated in schizophrenia. However, the postnatal development of these structures along with their associated neurons in the PFC is unexplored, as is their effects on behavior and neural activity. Therefore, in this study, we employed a DISC1 (Disruption in Schizophrenia) mutation mouse model of schizophrenia to assess these developmental changes and tested whether enzymatic digestion of PNNs in the PFC affected schizophrenia-like behaviors and neural activity. Developmentally, we found that the normal formation of PNNs, PVs, and colocalization of these two in the PFC, peaked around PND 22 (postnatal day 22). However, in DISC1, mutation animals from PND 0 to PND 60, both PNNs and PVs were significantly reduced. After enzymatic digestion of PNNs with chondroitinase in adult animals, the behavioral pattern of control animals mimicked that of DISC1 mutation animals, exhibiting reduced sociability, novelty and increased ultrasonic vocalizations, while there was very little change in other behaviors, such as working memory (Y-maze task involving medial temporal lobe) or depression-like behavior (tail-suspension test involving processing via the hypothalamic pituitary adrenal (HPA) axis). Moreover, following chondroitinase treatment, electrophysiological recordings from the PFC exhibited a reduced proportion of spontaneous, high-frequency firing neurons, and an increased proportion of irregularly firing neurons, with increased spike count and reduced inter-spike intervals in control animals. These results support the proposition that the aberrant development of PNNs and PVs affects normal neural operations in the PFC and contributes to the emergence of some of the behavioral phenotypes observed in the DISC1 mutation model of schizophrenia.

## 1. Introduction

Schizophrenia is a neurodevelopmental disorder whose clinical symptoms begin appearing from pre-adolescence to early adulthood [1,2]. Heritability of this disease is as high as 80%, indicating a high genetic predisposition [3,4,5,6,7,8], which, along with pre- and peri-natal environmental influences, converge onto molecular and structural perturbations in the brain [9,10,11]. Structural alterations in the prefrontal cortex (PFC), hippocampus and entorhinal cortex, among other brain regions, lead to positive, negative, and neurocognitive symptoms [12,13,14,15,16]. Although psychosis appears later in the lifetime trajectory [17,18,19], the cascade of pathological changes leading to this outcome starts during embryonic development [20,21].

An imbalance of excitatory and inhibitory (E/I) neural activity is associated with many neuropsychiatric disorders, including schizophrenia [22,23,24,25,26,27,28,29,30]. Among the neural substrates affected in schizophrenia, parvalbumin-positive interneurons (PVs), which are fast-spiking, inhibitory interneurons [31,32,33], and perineuronal nets (PNNs), which form part of the extracellular matrix (ECM) that surrounds PVs, both regulate E/I balance in the cortex [34]. PNNs vary spatially and temporally across species and individual brains [35,36,37] and maintain the ionic balance in the micro-environment of PVs [38,39,40]. During the closure of the critical period, coinciding with the stabilization of E/I circuitry, PNNs form into well-formed lattices surrounding a high percent of PV interneurons [41,42,43]. The unique environment of PNNs is determined by activity dependent secretion of the ECM by surrounding neurons and other cells [44]. These structures help maintain the ionic balance around neurons and act as physical barrier, playing a role in synaptic plasticity and signaling, binding to proteins such as otx2 and semaphorin [45,46].

Although aberrations in PNN and PV structure are implicated in schizophrenia, it remains unclear how the distribution and formation of PNNs progressively change during development and how these changes affect normal behavior and physiology [38,47,48]. ECM structures are also of potential therapeutic interest due to their role in development during the critical period and the maturation of neural circuitry [49,50]. In various animal studies, these structures have been enzymatically manipulated to enhance cognition and restore ‘juvenile-like’ plasticity of neural synapses [44,49,50]. The spatial and temporal abundance of PNNs is finely balanced to ensure the accurate formation of mature neural circuitry [50]. Untimely and indiscriminate formation or alteration of these structures can lead to negative consequences, due to reduced protection of neurons against reactive ion species and/or neurotoxins [50]. 

Consequently, in the present study, we assessed the pattern of development of PNNs related to PVs in the PFC of a genetic mouse model of schizophrenia (129SvEv), harboring mutations in the DISC1 (disrupted-in-schizophrenia) gene compared with controls at different age points. We further explored the behavioral and electrophysiological impact of enzymatic digestion of PNNs in the PFC of these animals. From these studies, we found anatomical, electrophysiological, and behavioral alterations associated with PNNs and PVs in the PFC, which we conclude underlie aspects of the clinical presentation of schizophrenia.

## 2. Results

### 2.1. Developmental Expression of PNNs and PVs in the PFC

Coronal sections from the prefrontal cortex (PFC) of mice at PNDs 15, 22, and 60 were quantitatively analyzed for WFA-labeled PNNs, PV-positive neurons (PVs) and their colocalization. We found diffuse expression of PNNs and PVs in the PFC from PNDs 0 and 8, which showed insignificant expression and changes of WFA positive (PNNs) and PV positive fluorescence at these ages, suggesting that these cells and the extracellular matrix are still in the process of forming stable structures [51]. PV expression was negligible and was not significantly colocalized with PNNs at PND 0, and at PND 8 [52]. 

**Figure 1:**
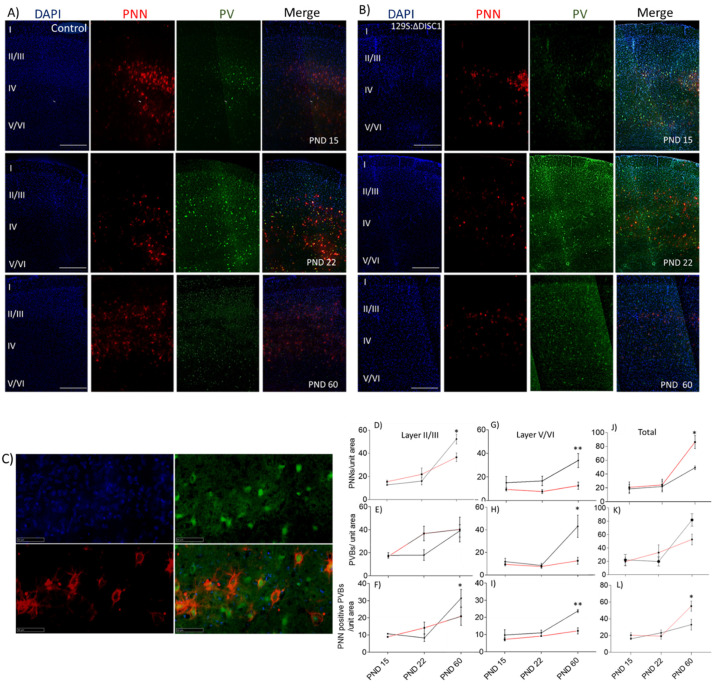
Representative immunofluorescent images and quantification**.** (**A**,**B**) control and DISC1 mutation**:** ∆DISC1 age wise representation from postnatal day (PND) 15, 22, and 60, respectively; (**C**) high magnification image showing colocalization of PNN (red)/PV (green) /DAPI (blue). Roman numerals I–VI indicate cortical layers 1–6, respectively; (**D**–**I**) quantitative analysis of PNN, PVs and colocalization of the two in layers II-VI. (**J**–**L**) Total PNNs, PVBs and colocalization in PFC layers Black and red lines indicate Control and DISC1 mutation animals, respectively. The data are represented as a scatter line plot showing mean ± s.e.m. where * *p* ≤ 0.05, ** *p* ≤ 0.01, by one way-ANOVA.

From PND 15 onwards, PNNs and PVs exhibited discrete expression patterns. Control animals showed a significant increase in well-formed PNNs in layer 2/3 from PND 22 to 60 (*p* < 0.0001) (Figure 1). Similarly, the number of PVs increased significantly from PND 22 to 60 (*p* = 0.0489 for layer 2/3 and at *p* = 0.0156 for layer 5/6). Colocalization of PV-positive neurons with PNNs also increased with age in both of the layer wise comparisons, exhibiting a significant increase from PND 22 to 60 (layer 2/3 at *p* = 0.0008; layer 5/6 at *p* = 0.0063), with an 82% and 73% of PVs colocalizing with PNNs in the layer 2/3 and 5/6 of PFC at PND 60, respectively. DISC1 mutation animals showed a significant rise in PNN positive PVs from PND22 to PND 60 (*p* = 0.043) (Figure 1B–H), with 42% and 96% of PVs colocalizing with PNNs in layer 2/3 and 5/6 of PFC, respectively.

When DISC1 animals were age-matched compared with controls, we found a significant reduction in PNNs at PND 60 in DISC1 animals (at *p* = 0.0463 in layer 2/3 and *p* = 0.0074 in layer 5/6 of PFC) (Figure 1A–C). Similarly, PVs in layer 5/6 were significantly reduced in DISC1 animals (at *p* = 0.0158) vs. control at PND 60. As such, colocalization of PNNs around PVs exhibited a significant reduction in DISC1 animals at PND 60 for layer 2/3 (at *p* = 0.0130) and 5/6 (at *p* = 0.0055), when compared to age-matched controls in these layers of PFC, respectively. These results support the changes in PNNs, PVs, and PNN-positive PVs across ages in both the str ains. These results are also indicative of relatively delayed and impaired growth of PNNs in the PFC of DISC1 animals.

### 2.2. Protein Expression

**Figure 2:**
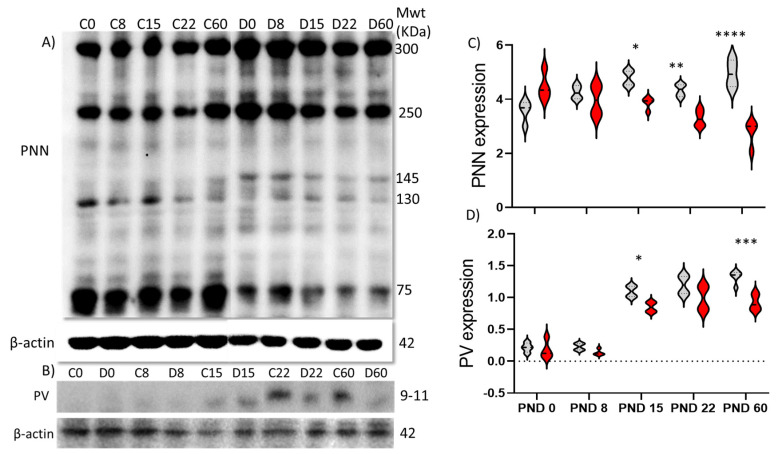
Age-wise expression of perineuronal net components. (**A**) perineuronal net components from PFC lysates (notations above the blots: *C* refers to control and *D* refer to 129S:ΔDISC1 at the respective postnatal ages in days); (**B**) parvalbumin band. Beta-actin expression was used as a loading control; (**C**,**D**) quantitative expression of PNN and PV, respectively. Black and red lines indicate of Control and DISC1 mutation animals, respectively. The data are represented as a scatter line plot showing mean ± s.e.m. where * *p* ≤ 0.05, ** *p* ≤ 0.01, *** *p* ≤ 0.001, and **** *p* ≤ 0.0001 by two way-ANOVA.

To further quantify expression of PNNs and PVs with age, we measured protein expression levels by Western blot from PFC lysates (Figure 2). We found that control animals exhibited an increased expression of PNNs from PND 0 to 8 (at *p* = 0.0016), followed by a decrease at PND 22 (at *p* = 0.0228) (Figure 2A,B), while PV expression showed a significant increase from PND 8 to PND15 (at *p* < 0.0001) (Figure 2). In comparison, DISC1 animals did not generally show a significant change in total PNN or PV expression with age (Figure 2A–D), except for a significant increase from PND 8 to 15 in PV expression (at *p* = 0.0044) (Figure 2B,D). These results match the changes in WFA-positive PNN expression as determined through the immunofluorescence studies described above.

When both strains were compared, we found that control animals had significantly higher PNN expression at ages PND15, 22 and 60 (at *p* = 0.0018, 0.019 and *p* < 0.0001 respectively), when compared to DISC1 animals of the same age (Figure 2A,B). The expression of parvalbumin in the PFC showed no significant difference among these two strains during early development but exhibited an overall significantly decreased expression at PND 60 (at *p* = 0.016; Figure 2C,D) vs. DISC1 mutation.

### 2.3. Chondroitinase Injections in PFC 

To test whether the difference in PNNs observed between control and DISC1 animals in the PFC may be a factor underlying differences in the two strains, we enzymatically digested PNNs using chondroitinase ABC. After injections of PFC with either chondroitinase or penicillinase (as a control), we assessed both strains of animals behaviorally, electrophysiologically, and neuroanatomically.

#### 2.3.1. Chondroitinase Injections Reduce the Number of Well-Formed PNNs

**Figure 3:**
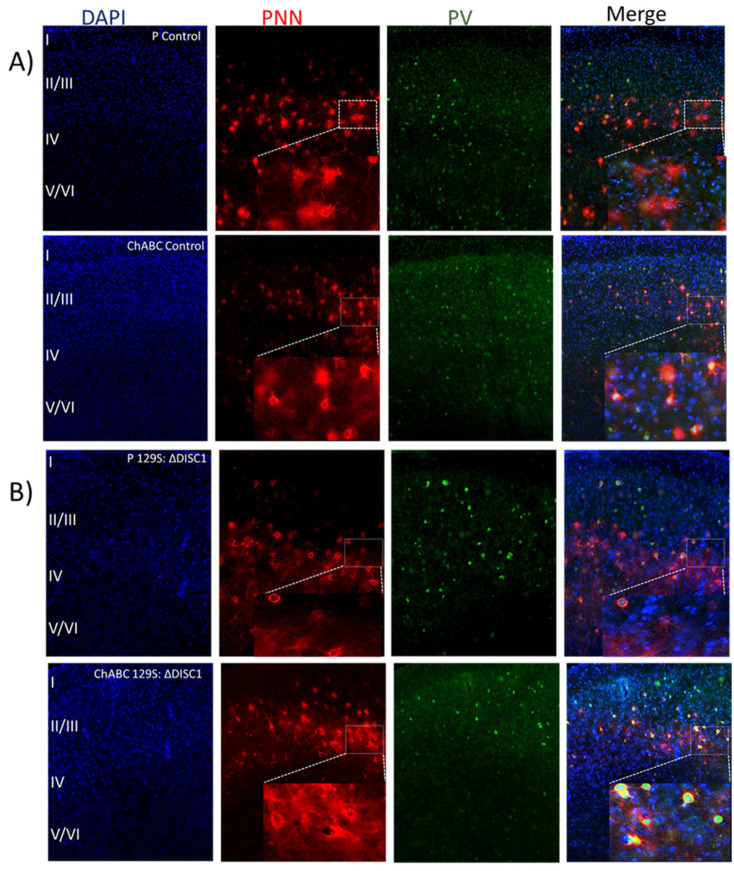

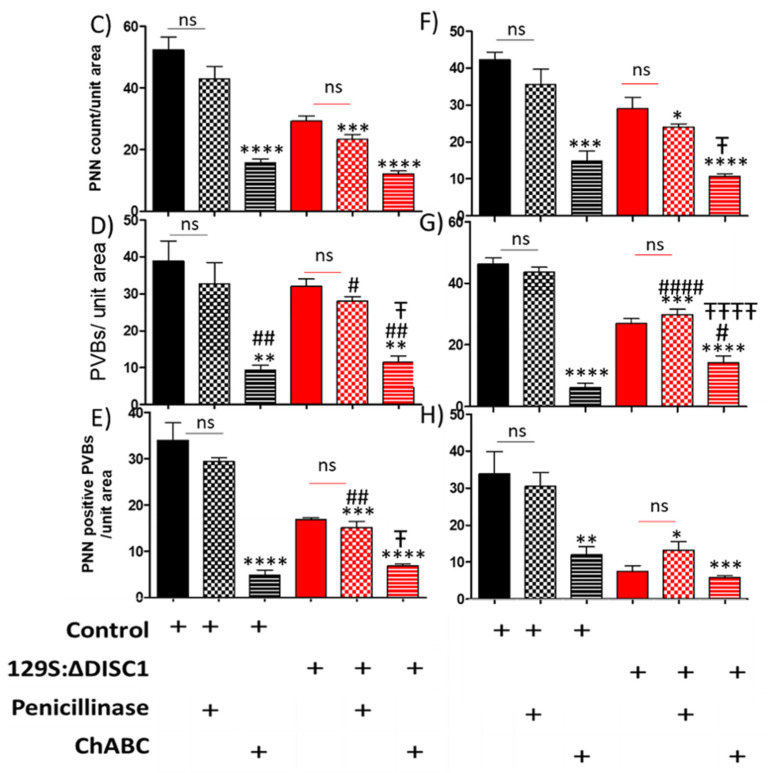
Analysis of immunofluorescence of chondroitinase and penicillinase treated animals PFC. (**A**,**B**) representative images of DAPI (blue), PNN (red), PV (green), and merged stains for animals treated at 8 weeks old. Panel A illustrates control animals treated with Penicillinase (P) or Chondroitinase (ChABC), while Panel B shows the results for 129S animals; (**C**–**E**) quantitative analysis of PNN, PV, and their colocalization in *layer 2/3* of PFC; (**F**–**H**) quantitative analysis of PNNs, PVs, and their colocalization in *layer* 5/6 of PFC. The top graphs show the density of PNNs (PNN count/area), the middle graphs indicate the density of parvalbumin neurons (PVB/unit area) and the bottom graphs indicate the density of colocalized PNNs with PVBs. The treatment groups are indicated in the *x*-axis legends below each column of graphs. Black and red bars represent control and DISC1 mutation background, respectively. The data are represented as plots showing mean ± s.e.m. where * or # *p* ≤ 0.05, ** or ## *p* ≤ 0.01, *** *p* ≤ 0.001 and **** or #### *p* ≤ 0.0001, where the symbols express the comparison of chDISC1 mutation animals with * represents comparison vs. penicillinase control, # represents comparison vs. chondroitinase control, and “Ŧ” represents comparison vs. Penicillinase treated DISC1.

Chondroitinase injections in the PFC of control and DISC1 animals were performed in 8-week-old animals and resulted in reductions to the distribution of well-formed (lattice-like) PNNs. To rule out any changes due to injection procedures, we also compared naive animals (control and DISC1) to animals injected with penicillinase (as an inert vehicle control) and found no significant difference between those groups (Figure 3A,B). We found that ChABC treated animals had significantly reduced numbers of PNNs versus those treated with penicillinase (control animals: layers 2/3 and 5/6 at *p* < 0.0001 and *p* < 0.0002, respectively; and DISC1 animals at *p* = 0.0154 in layer 5/6; Figure 6A,C). Interestingly, we also found a significant decrease in the number of PV-positive neurons in ChABC treated animals in layer 2/3 (at *p* = 0.0017 for ChABC treated-controls, and at *p* = 0.0312 for ChABC DISC1 animals versus the respective penicillinase treated animals; Figure 6A,B,D–G) and 5/6 (at *p* < 0.0001 for both ChABC treated control and DISC1 mutation animals vs. *p*-treated animals of both groups) of PFC (Figure 6A,B,D,G). Colocalization of PNNs with PVs also decreased in chondroitinase treated animals in both groups (layer 2/3 *p*-control vs. ChABC control and ChABC DISC1 at *p* < 0.0001 and *p*-DISC1 vs. ChABC DISC1 at *p* = 0.0375; Figure 6A,B,E–G).

Interestingly, control animals exhibited greater alterations than DISC1 mutation animals following chondroitinase injections, in terms of the structure and distribution of PNNs, parvalbumin interneuron count, and colocation of PNNs with PVs. This may be due to a significantly higher number of well-formed PNNs in controls vs. DISC1 animals (as shown in Figure 1A–C and Figure 6A–C). Chondroitinase treated DISC1 animals exhibited decreased numbers of PNNs, PVs, and PNNs surrounding PVs, but the percent of PVs surrounded by PNNs was unaffected (Figure 6).

#### 2.3.2. Chondroitinase Treated Animals Show Aberrant PFC Associated Behaviors

**Figure 4:**
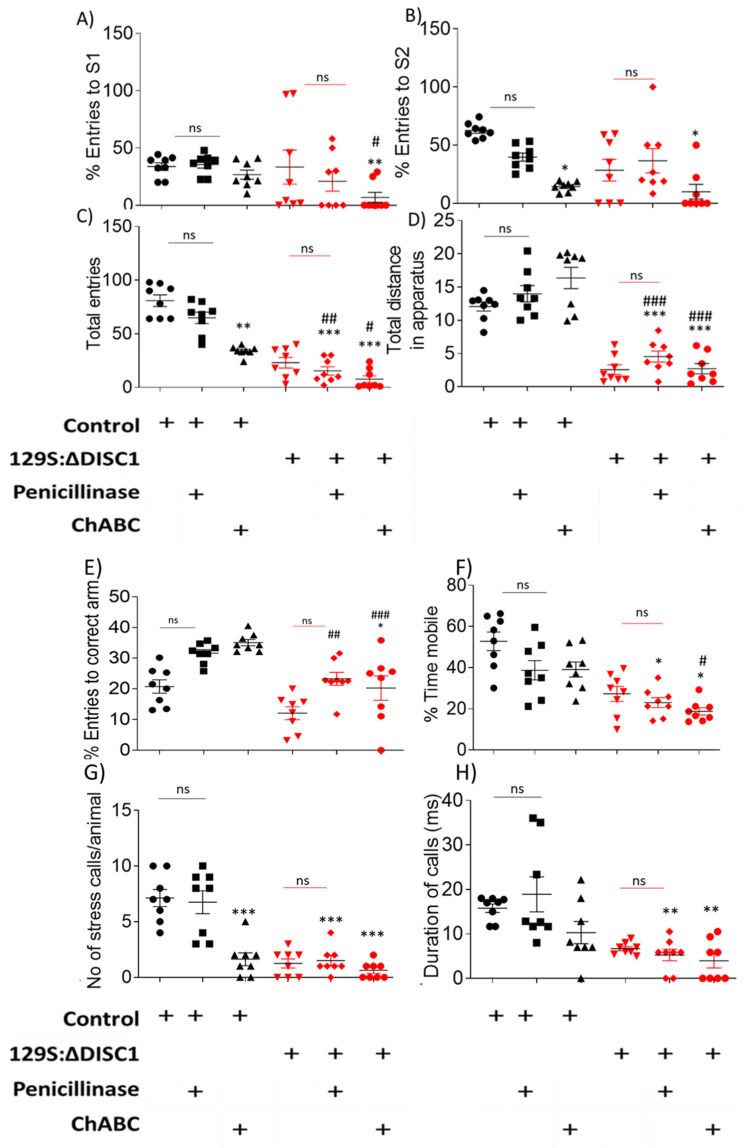
Behavioral analysis of chondroitinase and penicillinase treated animals. (**A**) sociability test as measured by the percent entries to the stranger 1 (S1) chamber; (**B**) novelty test as measured by the percent entries to the stranger 2 (S2) chamber; (**C**,**D**) The overall activity of the animals was assessed in terms of total entries from one chamber to another (*C*) and the total distance travelled throughout apparatus in meters (**D**). (**E**) In the Y-maze task, working memory was assessed by the percent entries into the correct arm of the maze; (**F**–**H**) stress responses were measured by the percent mobility time in a tail suspension test (**F**) and the stress calls produced during tail suspension test (**G**,**H**). Black and red symbols indicate Control and DISC1 mutation animals, respectively. The data are represented as plots showing mean ± s.e.m. where * or # *p* ≤ 0.05, ** or ## *p* ≤ 0.01, *** or ### *p* ≤ 0.001, where the symbols express the comparison of chDISC1 mutation animals with “*” represents comparison vs. Penicillinase control and “#” represents comparison vs. Chondroitinase control.

When tested for PFC associated behaviors, chondroitinase-treated control animals exhibited significantly reduced sociability versus penicillinase-treated control animals (*p* = 0.0356) and chDISC1 animals (*p* = 0.0001) (Figure 4A). Novelty was also reduced in the chondroitinase-treated control animals vs. penicillinase-treated control animals (*p =* 0.0106) and ChABCDISC1 animals vs. *p*-controls at *p* < 0.0001. ChABCDISC1 animals also showed significant differences from *p*-DISC1 animals at *p* = 0.0046, respectively; Figure 4B). Overall activity of these animals was also affected (Figure 4C,D). Surprisingly, we found that Chondroitinase treated control animal behavior was not significantly different from any of the DISC1 animal groups with (untreated or penicillinase), but a decrease in novelty vs. *p*-DISC1 group (*p* = 0.0046) (Figure 4A,B) (Table 1). These results suggest that the behavior of control animals following chondroitinase digestion of PNNs in the PFC is greatly affected, producing behaviors that resemble DISC1 mutation animals (Figure 4A,B). Although chondroitinase-treated DISC1 animals exhibited no significant PFC-related behavioral changes compared to untreated or penicillinase-treated DISC1 animals, the variance in behaviors was reduced among the chondroitinase-treated DISC1 animals (Figure 4A–D), suggesting a possible floor effect for these behaviors.

However, when tested for behaviors not directly related to the PFC, such as working memory and depression-like behaviors, we found no significant difference in the chondroitinase-treated animals vs. penicillinase-treated vs. untreated groups for all animal strains (Figure 4E,F). An exception to this was found in the tail suspension test, where we observed a significant difference in ultrasonic vocalizations in terms of the number of of stress calls (Figure 4G,H, P-control vs. ChABC-control and ChABC-DISC1 at *p* = 0.0002, and *p* = 0.0001, respectively, also shown in Table 1), but not in the duration of stress calls. Changes to the ultrasonic vocalization rate could possibly be due to the role of the PFC in emotional expression by the animals while under stress [53].

#### 2.3.3. Chondroitinase Treated Animals Exhibit Electrophysiological Changes in PFC

**Figure 5:**
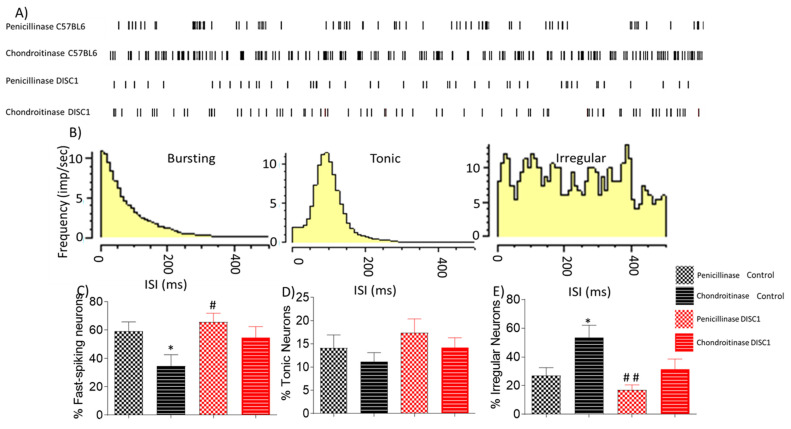
Electrophysiological classification of neural changes after chondroitinase or penicillinase treated animals as determined by extracellular recording of spontaneous neuronal activity in the PFC. (**A**) example firing patterns of spontaneously recorded neurons in different groups; (**B**) characteristic frequency for bursting, tonic, and irregularly firing neurons; (**C**–**E**) percent distribution of types pf neurons as recorded spontaneously from PFC of animals, i.e. fast spiking, tonic and irregularly firing neurons respectively. The data are shown as mean ± s.e.m. where * or # *p* ≤ 0.05, ## *p* ≤ 0.01, where the symbols express the comparison of chDISC1 mutation animals with * represents comparison vs. penicillinase control and # represents comparison vs. chondroitinase control.

To assess whether spontaneous neural activity in the PFC was altered following chondroitinase-treatment, we electrophysiologically recorded in vivo spontaneous activity. We found that digestion of PNNs in the PFC led to an overall increased firing rate in ChABC-treated animals, with the highest activity in ChABC treated-control animals (Figure 5A). The recorded neurons were further divided into categories on the basis of firing frequency (fast-spiking, tonic or irregular) (Figure 5B). We found that the proportion of fast-spiking neurons recorded was significantly reduced in ChABC-control animals vs. p-control animals (at *p* = 0.05), and there was a significant increase in the proportion of irregularly firing neurons recorded (*p*-control vs. ChABC-control at *p* = 0.0231), with no significant change in the proportion of tonic neurons (Figure 5D).

**Figure 6:**
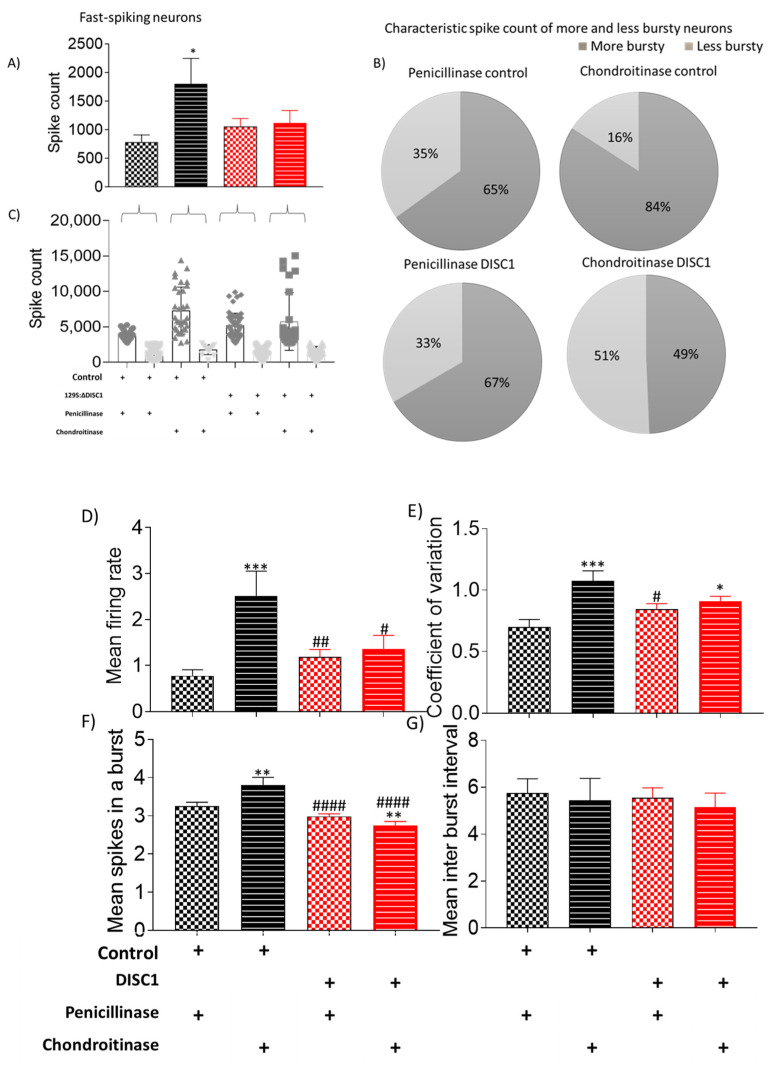
Characteristic of fast-spiking neurons. (**A**–**C**) spike count in recorded potentials and pie chart representing distribution of high and low spike count over and below threshold from respective groups; (**D**–**G**) exhibiting mean firing rate, coefficient of variation, and mean spikes in a burst and mean inter-burst interval, respectively. The data are shown as mean ± s.e.m. where * or # *p* ≤ 0.05, ** or ## *p* ≤ 0.01, *** *p* ≤ 0.001 and #### *p* ≤ 0.0001. * Represents comparison vs. penicillinase control and # represents comparison vs. chondroitinase control.

**Figure 7:**
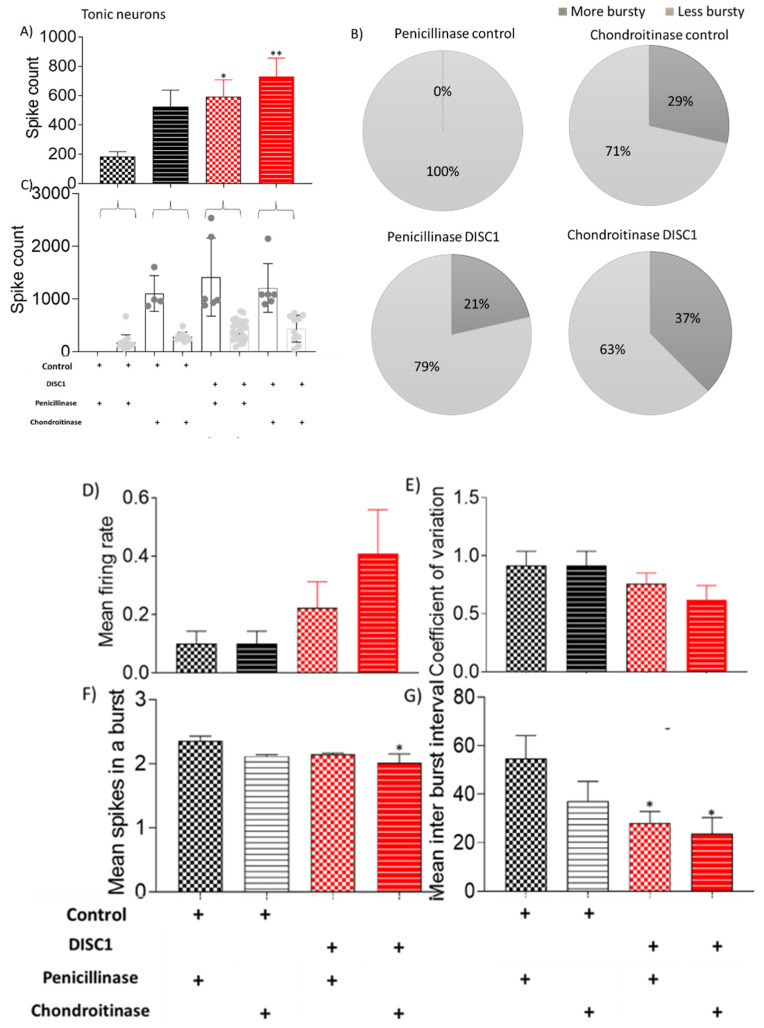
Characteristic of tonic neurons. (**A**–**C**) spike count in recorded potentials and pie chart representing distribution of high and low spike count over and below threshold from respective groups; (**D**–**G**) exhibiting mean firing rate, coefficient of variation, and mean spikes in a burst and mean inter-burst interval, respectively. The data are shown as mean ± s.e.m. where * *p* ≤ 0.05, ** *p* ≤ 0.01. * Represents comparison vs. penicillinase control.

**Figure 8:**
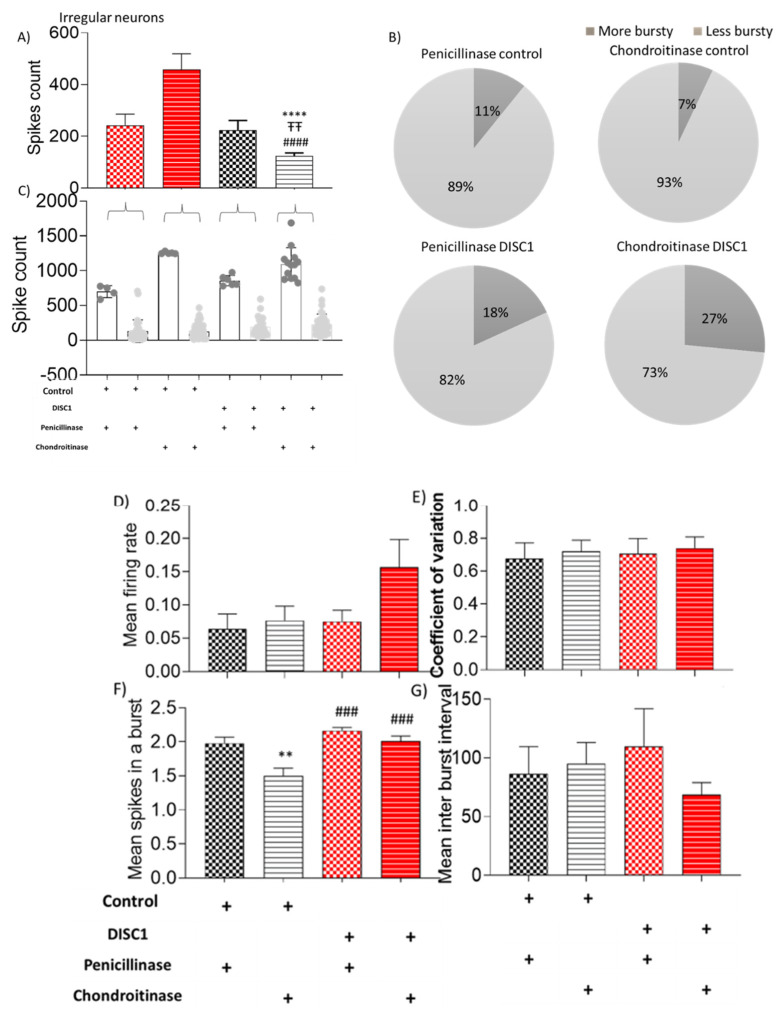
Characteristic of irregular neurons. (**A**–**C**) spike count in recorded potentials and pie chart representing distribution of high and low spike count over and below threshold from respective groups; (**D**–**G**) exhibiting mean firing rate, coefficient of variation, mean spikes in a burst, and mean inter-burst interval, respectively. The data are shown as mean ± s.e.m. where ** *p* ≤ 0.01, ###*p* ≤ 0.001 and **** or ####*p* ≤ 0.0001. * Represents comparison vs. penicillinase control, # represents comparison vs. chondroitinase control, and “Ŧ” represents comparison vs. Penicillinase treated DISC1.

These neurons as a group were further analyzed on overall spike rate (Figure 6, Figure 7 and Figure 8). The neurons were classified as high or low spiking, when analyzed with the mean spike count of the whole group as a threshold. We found that there was a significant increase in overall spike rate of ChABC-control animals (at *p* = 0.0059) and ChABC-DISC1 animals (at *p* = 0.0026) vs. *p*-control animals (Figure 5A and Figure 6A). When analyzed on the basis of overall spike rate, we found that ChABC-control animals exhibited an increase in higher spiking neurons as compared to p-control animals (showing an increase from 65% to 84%), whereas the ChABCDISC1 animals showed a decreased number of higher spiking neurons (Figure 6B).

The distribution of neuronal types recorded was similar in all DISC1 mutation animals (irrespective of treatment) and ChABC-control animals (Figure 6C: arrows). In comparison, ChABC-control animals exhibited a shift in neuronal firing rate when PNNs were digested in the recording site. However, DISC1 animals exhibited a balance of neurons firing at high and low rates, respectively (see Figure 6B,C).

Other properties of recorded neurons, such as mean firing rate, coefficient of variation, and mean spikes, were also significantly affected in ChABC treated animals of both groups (control and DISC1 mutation) vs. penicillinase-treated controls. Tonic neurons exhibited a significantly increased spike count in ChABC DISC1 mutation animals vs. penicillinase-controls (*p* = 0.0075, Figure 7A). When characterized on the basis of firing rate, there was an increased percent of high firing rate neurons in ChABC-control and ChABC DISC1 animals (29% and 37% vs. *p*-control (0%) and *p*-DISC1 (21%) respectively).

Mean spikes in a burst and inter-burst interval in ChABC DISC1 were also affected (*p* = 0.0127 and *p* = 0.0218 vs. p-control respectively; Figure 8F,G). Irregular spiking neurons show significantly increased spike count in ChABC DISC1 animals vs. p-control animals (at *p* < 0.0001, Figure 8A). When characterized further based on spike count, we found an increase in the high spike rate neurons in ChABC DISC1 animals vs. p-DISC1 (27% and 18%, respectively). There was a significant reduction in the mean spikes in a burst in ChABC control animals (*p* = 0.0058, Figure 8F), vs. *p*-DISC1 animals. These results indicate a significant change in the electrophysiological properties of chondroitinase treated animals from PFC recorded activity.

## 3. Discussion

In this study, we assessed a DISC1 mutation mouse model at postnatal days (PND 0, 8, 15, 22, and 60) compared with control animals to compare the growth pattern of PNNs, PVs, and PNN-associated PVs. We have previously characterized behavioral differences between these two strains, which were associated with the DISC1 mutation [54]. From the present results, we find significant variation in the development of PNNs and PVs in the PFC of DISC1 and control animals. In addition, we used chondroitinase to enzymatically digest PNNs and characterized the resultant changes in the expression of PNNs and PVs, correlated with corresponding behavioral and electrophysiological alterations. Overall, we found a differential expression of PNNs, PVs, and their colocalization as the animals aged, peaking at PND 15–22 in both strains. Digestion of PNNs with chondroitinase revealed neuroanatomical, behavioral, and electrophysiological alterations that suggest a role for these structures in the behavioral manifestation of schizophrenia.

### 3.1. Development of PNNs and PVs

The appearance and maturation of PNNs changes over time, with a differential expression of these ECM structures across brain regions [34,55,56,57,58]. In our study, we investigated PNN development during the critical postnatal developmental window from PNDs 0 to 60 [52,59,60]. We found that PNNs were scarcely formed from PND 0 to 8, lacking a well-formed lattice-like structure in both DISC1 and control strains (Figure 1). The diffuse appearance of WFA-positive staining could be due to the lack of galactose binding sites for *wisteria* lectins at these ages, which appears in well-formed PNNs at later ages [61]. The window for formation of well-formed PNNs also coincides developmentally with the closure of the critical period, neuron pruning, and synapse maturation in mice [62].

Parvalbumin neurons tightly control pyramidal cell output in the PFC, and thus are crucial for cognitive development [35,63]. Their dysregulation in the PFC has been associated with a disturbance of oscillatory network synchrony, linked with cognitive deficits in schizophrenia [64,65,66]. We found that parvalbumin-positive immunofluorescent staining was sparse at early ages (Figure 1), which was corroborated by Western blot analysis that showed very low to no expression of PVs from PND0 to 8. Positive expression for PVs was visible around prepubertal stages i.e., PND15, matching prior studies that examined PV cell maturation [35]. With the later co-appearance of PNNs, increased PV number could also be explained either by an actual low expression or an inability of these neurons to thrive due to lack of protection against oxidative stress from their higher metabolic activity. In these studies, we did not assess any potential colocalization with pyramidal neurons, which remains for future studies.

As the animals aged, PNNs began forming well-defined structures surrounding parvalbumin-positive interneurons, peaking around PND 22 to PND 60. However, compared with control animals, the formation of PNNs in DISC1 animals lagged temporally and was more diffuse throughout all the ages examined. The significant difference in expression of PNNs, PVs, and colocalization between the two groups suggested that these were a neural substrate underlying the PFC-related behavioral differences (sociability, novelty, working memory tasks) that these strains exhibit, as we have described previously [54]. These data also corroborate prior studies that indicate an important role of PNNs in the mPFC for the acquisition and consolidation of specific behaviors and memories [67]. The decreased number of PNNs and resulting PVs in DISC1 mutation animals could also contribute to the aberrant firing pattern of neurons, lacking synchrony in the PFC of schizophrenic individuals [68,69]. The DISC1 mutation could impact expression of IGF-1R (Insulin Like Growth factor-1 receptor), ECM (extracellular matrix) receptors, and related kinases on the neuronal surface, which acts as an anchor for PNNs [70]. The resulting loss of PNN protection around PVs may lead to their decreased number, as indicated by the decreased number of PVs colocalizing with PNNs (Figure 1 and Figure 2).

### 3.2. Chondroitinase Treatment of PNNs in PFC

Due to the neuroanatomical differences in the development and expression of PNNs and PVs in DISC1 and control animals (Figure 1, Figure 2 and Figure 5), we tested the effects of enzymatic digestion of PNNs on the expression of these structures, behavioral phenotype, and neural activity in the PFC [71,72]. We found that, following chondroitinase digestion, PNN count decreased in ChABC treated animals of both strains (Figure 4). PV interneurons were also reduced in these animals, which may be a result of increased oxidative stress on these neurons in the absence of well-formed PNNs. This is supported by our results that demonstrate, although the number of PVs and PNNs were reduced following digestion, that the percent colocalization did not change significantly within strain (in DISC1 mutation 50% in both untreated and treated). We also noticed a greater effect of chondroitinase digestion in the control animals (PNN count decreasing from 52 to 15 per unit area in PFC; Figure 4C), possibly due to their initially significantly higher number of well-formed PNNs compared with DISC1 animals. We speculate that this may reflect different PNN types in the PFC, with some more structurally resilient than others.

Behaviorally, we found that control animals treated with chrondroitinase exhibited significantly altered PFC related phenotypes, i.e., impaired sociability and novelty [73,74,75], when compared with untreated or penicillinase-treated groups. Interestingly, the behavior of chondroitinase-treated control animals was not significantly different from any of the DISC1 groups (untreated DISC1, penicillinase-treated, or ChABC treated groups). Chondroitinase treatment may thus produce a neuroanatomical state in control animals that mimics DISC1 animals, which have fewer well-formed PNNs and PVs. In addition, these results suggest that structural aberrations of PNNs and PVs could be a causative factor towards the altered behavioral display in afflicted individuals or in animal models of schizophrenia. 

In addition, we speculated that neural activity would be affected by PNN digestion, which would deplete the protective covering around PVs, affecting their survival and firing pattern [34]. PVs exert a strong inhibitory control over excitatory pyramidal cells, consequently regulating E/I balance in the cortex [76]. Our findings revealed that fast-spiking neuronal activity decreased significantly in the chondroitinase-treated control animals (from 60 to 30 percent) (Figure 5C), with a corresponding increase in irregularly firing neurons (from 26 to 52%) (Figure 5E). We deduce that the reduced number of fast-spiking neurons corresponds to the decreased PV-positive neuron counts found during immunofluorescent studies, which exhibit a similar reduction (see Figure 4). 

Interestingly, when each class of neurons was analyzed individually, increased firing rates appeared to emerge following ChABC-treatment. For instance, although the number of fast-spiking neurons decreased, the overall frequency of spiking in this neuronal population increased along with a reduction in inter-spike intervals (Figure 6). In the ChABC-treated control group, a new sub-group of fast-spiking neurons emerged that showed a very high spike rate (Figure 6C), which was absent in penicillinase-treated control animals. Interestingly, tonic and irregular firing neurons also exhibited some significant increases in firing rate following ChABC-treatments (Figure 7 and Figure 8). Such alterations to overall firing rate are consistent with a broad dysregulation of E/I network balance following PNN disruption.

Our data also suggest that the loss of PNNs around PV neurons alter their firing properties, potentially converting fast-spiking neurons to irregular spiking neurons (Figure 5). Loss of fast-spiking neurons also corresponds to the loss of PNNs when immunofluorescent data are considered, where PNNs were reduced from 42% in P-control to 15% in chondroitinase-treated control animals (data not shown). These findings could account for the E/I imbalance and loss of synchrony in the PFC and thus the resultant cognitive performance alterations in the DISC1 mice, which have an inherent loss of PNNs (Figure 1) or in control animals after chondroitinase treatment (Figure 4 and Figure 5). However, our present study did not attempt to directly assess such a conversion, such as by assessing changes to intrinsic membrane properties of neurons in response to chondroitinase treatment. A future avenue for investigation then could be to assess how such chemically, electrophysiologically, and morphologically defined neuronal subtypes are differentially affected by PNN degradation.

Overall, these data are consistent with a decrease in the number of well-formed PNNs in the PFC following degradation with ChABC. However, it is important to note that this enzyme not only affects the CSPG-CS bond, but also has a non-specific action on other ECM structures that share CS bonds, such as dermatan sulphate and hyaluronic acid [77]. Prior studies have demonstrated the effects of preferential ChABC digestion of PNNs, also due to the abundance of CSPGs when compared to other components of ECM [78]. However, further investigations are still required to determine the extent of its non-specific action as well as the relationship of PNN and PV structures in other schizophrenia-related behaviors.

## 4. Materials and Methods

All experiments were conducted according to NIH guidelines and were approved by the Institutional Animal Care & Use Committee (IACUC) of the Louisiana State University School of Veterinary Medicine. Animals were obtained from the Jackson Laboratory (Bar Harbor, ME, USA) and LSU DLAM. Mice from PNDs 0, 8, 15, 22 and 60 (*n* = 12 from each group, *n* = 6 males, and *n* = 6 females) were characterized developmentally. A total of 10 animals were used for enzymatic digestion experiments in each group (*n* = 5 males and *n* = 5 females). Equal numbers of male and female mice were examined separately for all assays; however, no significant differences were found; therefore, results are presented with genders combined. DISC1 mutation (129SvEv: ∆disc1) and C57Bl6J (control) groups (from here on referred to as DISC1 and control respectively) were used for these studies, as previously characterized in our prior studies [54,79]. Animals were housed in a temperature and humidity-controlled room with a 12 h light/dark cycle with lights on at 7:00 a.m. and food and water provided ad libitum. 

### 4.1. Histological Staining and Imaging

Brains were collected from pups (*n* = 6) at PNDs 0, 8, 15, 22, and 60. Animals were anaesthetized using isoflurane anesthesia, followed by intracardiac perfusion with 0.01 M PBS (maintained at 4 °C), followed by perfusion with 4% PFA (paraformaldehyde) in 0.01 M PBS for fixation. Brains were extracted and post fixed in 4% PFA in 0.01 M PBS overnight, then cryoprotected in a 30% sucrose/4% PFA solution in 0.01 M PBS. After 2–3 days, brains were then sectioned coronally at 40 m on a cryostat and collected in micro-well plates.

Three sections per animal (*n* = 6 each group) were imaged. A 1:6 series of sections were double labeled for both PNNs and PVs. To label PNNs, Biotinylated WFA (*Wisteria floribunda agglutinin* lectin) (L-1350) (Vector Labs, Burlingame, CA, USA) was used at a dilution of 1:1000. To label parvalbumin interneurons, a rabbit polyclonal anti-parvalbumin antibody (ab11427) (Abcam, Cambridge, UK) was used at a dilution of 1:500. Following an overnight incubation at 4 °C with either WFA or PV primary antibody, the sections were then washed three times (5 min each) in 0.01 M PBS. For PNNs, conjugated streptavidin Alexa Fluor 568 (Biotium CF-29035) and for PVs goat anti-rabbit Alexa Fluor 488 (ab150081) were used at a dilution of 1:5000 for a period of 60 min followed by three washes (5 min each) with 0.01 M PBS. The sections were mounted on gelatin subbed slides with mounting media for fluorescence containing DAPI (Vectashield H-1400) (Vector Labs).

For imaging, a NanoZoomer S60 digital slide scanner (C13210-01) (Hamamatsu, Bridgewater, NJ, USA) was used for obtaining images at 40×, and higher magnification (60×) images were obtained using a Nikon-*NiU* fluorescence upright microscope (Nikon, Melville, NY, USA). Labeling was analyzed to quantify number of PNNs and PVs (for PND 15, 22, and 60).

Quantification was completed on unmodified images by an observer blind to the specific experimental conditions of tissue analyzed. Cell counts for DAPI+ and PV+ cells were all counted using the Image-based Tool for Counting Nuclei (Centre for Bio-image Informatics, UC Santa Barbara, CA, USA) plugin for NIH ImageJ software. Within a target region, a standard total area was measured over the region of interest within which cells were identified and cell counting parameters kept constant. PNNs were counted manually using the ImageJ Cell Counter function within a standard total area over the target region. For each stain and each region, measurements of mean brightness within the area were also taken. Measurements and counts for each brain region are the average of 3 images taken from 3 adjacent sections. Age- and strain-wise comparisons were made using one-way and two-way ANOVA. Tukey and Bonferroni’s post-hoc tests were used respectively. The results are presented as bar graphs and/or scatter line plots representing mean ± s.e.m. 

### 4.2. Western Blotting for Protein Expression

In animals used for Western blot and protein analysis, brain tissue was extracted, and the location of PFC determined according to a standard atlas [80]. The PFC was then dissected, homogenized, and analyzed to determine total protein concentration using a Bradford assay. Samples (10 μg protein) were electrophoretically separated on a polyacrylamide gel (8% for PNNs and 12% for PVs), and then a wet-transfer method to a PVDF membrane. The membrane was then washed with 0.1% Tween-20 in 1× TBS (i.e., 1× TBST), followed by blocking with 3% BSA for an hour at RT, and incubated to detect PNNs or PVs as follows. To determine expression of PNNs and its components, membranes were incubated with biotinylated WFA (Vector Labs L-1350) at a dilution of 1:1000, while GAR-anti Parvalbumin (ab11427) at dilution of 1:1000 was used to detect PV expression incubate at 4 °C overnight. After overnight incubation, the membranes were washed in 0.1% Tween-20 (in 1× TBS) three times for five minutes each, followed by incubation with the HRP-conjugated streptavidin-biotin-HRP (for PNNs) or HRP-conjugated GAR (goat anti-rabbit) secondary antibody at 1:10,000 dilution (for PVs) for 1 hr at room temperature. The membranes were then washed with 0.1% Tween 20 in 1× TBST, three times for five minutes each. 

For imaging, chemiluminescent developing reagent was used (Pierce 32106) (Thermo-Fisher Scientific, Waltham, MA, USA). For loading standardization, the same membranes were stripped using Restore PLUS Western Blot Stripping Buffer (Thermo-Fisher Scientific #46430) and incubated with a mouse monoclonal, HRP-conjugated, beta-actin antibody (8H10D10) (Cell Signaling #12262S) for one hour at RT, then developed with the chemiluminescent reagent. The protein per lane was then normalized with the β-actin concentration of the respective lane. All the results obtained were repeated at least twice for all proteins. The data are presented as a scatter line plot comparing mean ± s.e.m, for age-wise within control animals or DISC1 mutation animals and between groups (control vs. DISC1 mutation, age-matched) using the statistical methods described below.

### 4.3. Chondroitinase ABC (chABC) Treatment of PFC (Adult Animals)

Protease-free ChABC (chondroitinase and ChABC used interchangeably; Amsbio, Seikagaku, Japan) or penicillinase (p; Spectrum, TCI, USA) as a control enzyme (with no known substrate in the brain) was dissolved in 0.1% BSA to 50 U/mL of concentration and filtered through a 0.2-micron filter [71]. In these experiments, 8-week-old animals (*n* = 5 males and females each) were used. Four injections in the PFC (2 per hemisphere, 0.25 μL with a speed of 0.2 μL/min) were performed stereotaxically under xylazine-ketamine injectable anesthesia (at 30 mg/kg) with a 10 μL Hamilton syringe and a 33 gauge needle at the following sites (in mm from bregma): anterior-posterior (AP): +2.68 mm; lateral (ML): ±1 mm; ventral (V): −2 mm and −1 mm. The needle remained in place at the injection site for 3 min between injections and an additional 3 min before being slowly withdrawn over a 2-min period. An *n* = 6 was used for each group (with 3 males and 3 females). Animals were returned to their home cages and were observed for a week before behavioral testing. 

### 4.4. Behavioral Testing

To determine the effects of PNN digestion on PFC related behaviors, animals were assessed on a sociability and novelty task, as previously described [54]. The Y-maze working memory task was used to ascertain influence on the PFC-hippocampus circuitry [81,82]. The tail suspension test and stress calls were evaluated to control for changes in other brain regions not expected to be influenced by chondroitinase ABC-treatment in the PFC [83,84].

### 4.5. In Vivo Electrophysiology in PFC of Chondroitinase (ChABC) and Penicillinase (p) Treated Animals

In vivo electrophysiological recordings from the PFC were performed in anaesthetized animals (using a xylazine and ketamine cocktail at 30 mg/kg). The plane of anesthesia was determined by assessing toe withdrawal reflex. The head of animal was affixed on stereotaxic apparatus and skull bone above the PFC region was removed using a drill bit (Dremel) to expose dura. ACSF (artificial cerebrospinal fluid) was used to prevent dryness of the exposed area. The injection (for either ChABC or P) sites were located, under a digital dissection microscope, and dura was carefully removed with the help of a sterile customized bent needle to expose the brain surface for electrode placement. An acute neural probe (Neuronexus, MI, USA) was used, whose shank was a tetrode carrying electrodes at an inter-electrode interval of 25 μm. The electrodes were connected to a 32-channel preamplifier head stage (Intantech, Los Angeles, CA, USA), linked to a 512-channel recording controller and amplifier system (Intantech). The probe was gently lowered using an ultrafine hydraulic micromanipulator (Narishige, Amityville, NY, USA) to reach the PFC at stereotaxic coordinates (AP: −2.68 mm, ML: +1.0 mm, DV: +2 and +1 mm) relative to the bregma (Franklin and Paxinos, 2008). Stainless steel ground wires soldered onto the head stage-electrode adapter (Neuronexus; A4 to Omnetics CM32 adapter) were tied to a ground screw that was fixed in the parietal bone, as detailed previously [85]. 

Single-unit activity was monitored for at least 20 min before recording began and spontaneously evoked data were obtained continuously for 45 min. Acquired neural recordings were then processed in offline-spike sorting software (Version 4.4.0; Plexon Inc., Dallas, TX, USA; RRID:SCR_000012). Further analysis of the sorted spikes was done in NeuroExplorer Version 5.121 (Nex Technologies, Fairfax, VA, USA; RRID:SCR_001818).

### 4.6. Neural Spike Processing and Analysis

Neural spikes were extracted from the continuous data through threshold crossing in the Off-Line Spike Sorter (OFSS). The extracted spikes were sorted using an unsupervised valley-seeking method and K-means clustering in three-dimensional (3D) PCA space. Where necessary, unsorted spikes were assigned to clustered units, or invalidated if outlying. Sorted neural spike waveforms, clustered units, and continuous data were exported into the NeuroExplorer software for further analysis. Continuously recorded data from at least two electrode units were considered per mouse. In the NeuroExplorer platform, interspike interval (ISI), peri-event rasters, and burst analysis histograms were plotted for the sorted spikes, as is also described in our previous work [86]. Neurons were divided based on regularity, spike-rate, tonicity, and type. Neurons were classified as fast-spiking, tonic, or irregular firing based on the binned distribution of their firing rate and interspike interval spread. By constructing an ISI histogram in Neuroexplorer, neurons with fast-spiking activity exhibited high event scores followed by a rapid decay by 100 ms. Tonic neurons were characterized by a phase without activity followed by a sharp rise in activity and then a decay. Irregular neurons were classified by skewed ISI histograms. The ISIH generally described the firing pattern and regularity of the putative neurons. Further statistical analysis was carried out on the characteristics based on frequency of firing, coefficient of variation, and interspike intervals.

### 4.7. Statistical Analysis 

One-way or two-way ANOVA was carried out as mentioned in respective methods and figure legends. Post-hoc tests, Tukey’s (one-way ANOVA), and Bonferroni’s (for two-way ANOVA) were used respectively. The graphs are represented as scatter line plots or bar graphs, depicting mean ± s.e.m.

## 5. Conclusions

In conclusion, we found that there was an increase in the expression of WFA-positive PNNs, PV-positive neurons, and their colocalization as animals developed (PND15 to PND 60 and adults, Figure 1 and Figure 5). Chondroitinase-treatment for PNN digestion decreased the number of well-formed PNNs and associated PVs (Figure 5), indicating the role of PNNs as a crucial factor in supporting the normal functioning of parvalbumin interneurons (Figure 7). However, it remains to be determined if other types of neurons are also affected by the induced change in PNNs in PFC, perhaps through the application of acute slice physiology and neuron-specific staining. Further probing of the cell-type specific role of PNNs might be elucidated through optogenetic manipulation of PV interneurons in combination with chondroitinase treatment [87,88]. Such an approach could enable a disentangling of PNN-specific versus network-mediated effects. Overall, our study delineated the effects of PNN growth over discrete postnatal days and illuminates the general effects of PNN digestion on behavioral outcomes and electrophysiological properties of PFC neurons.

## Figures and Tables

**Table 1:** Summary of the behavioral tests. All results are shown as compared to a penicillinase-control group.

S. No.	Behavioral Test	Vs. ChABC Control	Vs. ChABC 129s:ΔDISC1
1	Sociability	Yes (*p* = 0.0356)	Yes (*p* = 0.0001)
2	Novelty	Yes (*p* = 0.0106)	Yes (*p* = 0.0001)
3	Y maze	No	Yes (*p* = 0.05)
4	Tail Suspension test	No	Yes (*p* = 0.03)
5	Stress calls	Yes (*p* = 0.0002)	Yes (*p* = 0.0001)

## Data Availability

All primary data from this study are available upon request to the corresponding author.
